# *LncRNA NEAT1* regulated diabetic retinal epithelial-mesenchymal transition through regulating miR-204/SOX4 axis

**DOI:** 10.7717/peerj.11817

**Published:** 2021-07-23

**Authors:** Yang Yang, Jing Zhou, Wei hong Li, Zhi xiong Zhou, Xiao bo Xia

**Affiliations:** 1 Eye center of Xiangya Hospital, Central South University, Changsha, Hunan Province, China; 2 Department of Ophthalmology, the First People’s Hospital of Yueyang, Yueyang, Hunan, China; 3 Hunan Key Laboratory of Ophthalmology, Central South University, Chang sha, Hunan, China

**Keywords:** Diabetic retinopathy, Epithelial-mesenchymal transition, NEAT1, miR-204, SOX4

## Abstract

**Aim:**

Epithelial-mesenchymal transition (EMT) of retinal pigment epithelium (RPE) cells is the key of the development of diabetic retinopathy (DR), and lncRNA NEAT1 could accelerate EMT in diabetic nephropathy. Meanwhile, as a diabetes susceptibility gene, whether sex-determining region Y-related (SRY) high-mobility group box 4 (SOX4) has relationship with lncRNA NEAT1 in DR remains unclear.

**Methods:**

Firstly, NEAT1, SOX4 and miR-204 were evaluated by qRT-PCR (quantitative reverse-transcriptase PCR) under high glucose condition. Then, cell viability, proliferation, migration and invasion were respectively detected by MTT, BrdU staining, wound healing and transwell assay after NEAT1 knockdown or miR-204 overexpression. Also, the EMT-related proteins were examined by western blot and cell immunofluorescence assay. In order to confirm the relationship between miR-204 and NEAT1 or SOX4, dual luciferase reporter gene assay was conducted. At the same time, the protein levels of SOX4 and EMT-related proteins were investigated by immunohistochemistry *in vivo*.

**Results:**

High glucose upregulated NEAT1 and SOX4 and downregulated miR-204 in ARPE19 cells. NEAT1 knockdown or miR-204 overexpression inhibited the proliferation and EMT progression of ARPE19 cells induced by high glucose. NEAT1 was identified as a molecular sponge of miR-204 to increase the level of SOX4. The effect of NEAT1 knockdown on the progression of EMT under high glucose condition in ARPE19 cells could be reversed by miR-204 inhibitor. Also, NEAT1 knockdown inhibited retinal EMT in diabetic mice.

**Conclusion:**

NEAT1 regulated the development of EMT in DR through miR-204/SOX4 pathway, which could provide reference for clinical prevention and treatment.

## Introduction

As a serious and destructive complication of diabetes, the prevalence of diabetic retinopathy (DR) is about 28.5%, which is the most common reason of blindness in adults of western nations (Solomon et al., 2017). The DR progression goes through several different pathological stages, gradually transitioning from non-proliferative diabetic retinopathy (NPDR) to proliferative diabetic retinopathy (PDR). At present, there is no effective intervention for vision recovery in patients with severe PDR (Gangaputra et al., 2013). During the onset of DR, retinal pigment epithelial cells (RPEs) may have undergone epithelial-to-mesenchymal transition (EMT) when exposing to high glucose environment (Saika et al., 2009). The upregulation of mesenchymal markers was obvious in RPE cells induced by high glucose (Che et al., 2016). Moreover, hyperglycemia increased advanced glycation end-product (AGE) formation and in turn exacerbated the pathogenesis of eye diseases, because AGE mimetic was demonstrated to breakdown the function of RPE cells (Dahrouj et al., 2015).

Many genetic variants found by genome-wide association studies (GWAS) increased the possibility of type 2 diabetes (Flannick & Florez, 2016), such as sex-determining region Y-related (SRY) high-mobility group box 4 (SOX4), which was discovered as a diabetes susceptibility gene and a new-called master regulator of EMT (Ragvin et al., 2010). It was reported that inhibition of SOX4 could decrease the expression of EMT-related mRNA expression and protein levels in human peritoneal mesothelial cells (HPMCs) induced with TGF-β1 (Xiao et al., 2015). However, the molecular mechanism of SOX4 in the progression of EMT in DR remains unclear.

Long non-coding RNA (LncRNA)-related biological activities were associated with many diseases. LncRNA nuclear-enriched abundant transcript 1 (NEAT1) was reported to promote the progression of EMT in diabetic nephropathy (Wang et al., 2019). However, little is known about the NEAT1 influence in DR. LncRNA could act as sponge to bind with microRNA (miRNA) to block its participation in various biological processes. NEAT1 was reported to be involved in human retinoblastoma cells by regulating miR-204 (Zhong et al., 2019). However, studies on the relationship between NEAT1, miR-204, and SOX4 in the progression of EMT in DR have not yet been reported.

Therefore, this study intended to investigate whether NEAT1 regulated high glucose-induced EMT through miR-204/SOX4 axis. An *in vitro* cell model demonstrated that high glucose upregulated NEAT1 and SOX4, downregulated miR-204 and exacerbated cell proliferation and the progression of EMT in ARPE19 cells. Then the influences of NEAT1 knockdown and miR-204 overexpression on the high glucose induced EMT in ARPE19 cells were verified, and the relationships among NEAT1, miR-204, and SOX4 were evaluated. Finally, the effects of NEAT1 on the retinal tissue of diabetic mice were further confirmed *in vivo*. We proposed that NEAT1 could regulate the progression of EMT in DR through the miR-204/SOX4 pathway.

## Materials & Methods

### Cell culture and treatment

Human retinal pigment epithelium (hRPE) cell line ARPE19 was obtained from ATCC (Manassas, VA, USA) and cultured in Dulbecco’s modified Eagle’s medium (DMEM, Invitrogen, Carlsbad, CA, USA) with fetal bovine serum (FBS, 10%, Invitrogen, Carlsbad, CA, USA), L-glutamine (0.292 mg/mL; Invitrogen, Carlsbad, CA, USA) and penicillin (100 U/mL; Invitrogen, Carlsbad, CA, USA), and replaced every 3 days. The culture condition was kept at 37 °C with 95% humidity and 5% CO_2_. After 95% confluence assessed by microscopy, cells were subjected to trypsin-EDTA digestion and used for experiments. 5.5 mM glucose was used in control group, and in high glucose group the concentration of glucose was 30 mM (Shi et al., 2015). An inverted microscope (Leica, Wetzlar, Germany) was used to observe the cell morphology.

### Quantitative reverse-transcriptase polymerase chain reaction (qRT-PCR)

ARPE19 cells or retinas of diabetic mice was applied with Trizol reagent (TransGen Biotechnology Co., Ltd., Beijing, China) to collect total RNA. Then, a cDNA Reverse Transcription kit (TransGen Biotechnology Co., Ltd., Beijing, China) was used to obtain cDNA. The amplified cDNA was acquired by TaqMan PCR Master Mix (TransGen Biotechnology Co., Ltd., Beijing, China). The primer sequences were showed in Table 1. GAPDH served as an internal reference gene for NEAT1 and SOX4, while U6 served as an internal reference gene for miR-204. According to the formula 2 ^−ΔΔCt^, the relative fold change of expression was calculated (Livak & Schmittgen, 2001).

**Table 1:** Primer sequences used in qRT-PCR.

Gene	Primers
miR-204(human)	5′-GGGCTTCCCTTT GTCATCCT-3′ 5′-GTGCAGGGTCCGAGGT-3′
miR-204(mouse)	5′-TATAGAATTCGCTTCATTCAGCACCTAGTT-3′ 5′-TATACTCGAGTGGGTAAGGTTCTTTGATGT-3′
SOX4 (human)	5′-GTGAGCGAGATGATCTCGGG-3′ 5′-CAGGTTGGAGATGCTGGACTC-3′
SOX4 (mouse)	5′-TCCCACTTCGAATTCCCGGACTAT-3′ 5′-AAGACCAGGTTAGAGATGCTGGAC-3′
NEAT1(human)	5′-GAGTTAAGGCGCCATCCTCA-3′ 5′-AGCACTGCCACCTGGAAAAT-3;
NEAT1(mouse)	5′-GGGGCCACATTAATCACAAC-3′ 5′-CAGGGTGTCCTCCACCTTTA-3′
U6	5′-GCTTCGGCAGCACATATACTAAAAT-3′ 5′-CGCTTCACGAATTTGCGTGTCAT-3′
GADPH	5′-CATCACCATCTTCCAGGAGCG-3′ 5′- TGACCTTGCCCACAGCCTTG -3′

**Notes.**

SOX4 SRY-related high-motility group box 4 NEAT1 nuclear-enriched abundant transcript 1 GADPH Glyceraldehyde-3-phosphate dehydrogenase

### MTT assay

ARPE19 cells at 5,000 cells/mL were culture in 96-well plate for MTT assay. After reaching 60% confluence, serum-free DMEM was applied. After 0, 24, 36 and 72 h incubated with serum-free DMEM and high glucose (30 mM), MTT (3-(4,5-dimethyldiazol-2-yl)-2,5-diphenyl tetrazolium bromide, Sigma-Aldrich, St Louis, MS, USA) at the concentration of 0.5 mg/mL was applied to each well of 96-well plate and subsequently cultured for 4 h. After applying dimethylsulfoxide (DMSO; Sigma-Aldrich, St Louis, MS, USA), a plate shaker was used for 5 min agitation. A purple-red solution was obtained with a maximum absorption value at 490 nm, which can reflect the number of viable cells. Therefore, the optical density of each well at 490 nm was measured immediately on an enzyme-linked immunosorbent assay reader (model MR 700; Dynatech Labs, Guernsey, UK) according to the manufacturer’s instruction.

### BrdU immunofluorescence staining

ARPE19 cells at 10^6^ cells/mL were cultured in slices which used for BrdU immunofluorescence staining. After specific treatment, cells were incubated with 10 µm BrdU solution for 90 min. After fixing and DNA denaturation, 5% bovine serum albumin (BSA; Thermo scientific, Waltham, MA, USA) was used for blocking, and primary antibodies BrdU were incubated over night. A fluorescent secondary antibody and DAPI were respectively incubated. Using anti-fluorescence quencher, the fluorescence pictures were taken from A fluorescence microscope. By analysing the red spot, cell proliferation activity was verified.

### Western blot

Total protein from ARPE19 cells or retinas of diabetic mice was harvested by Protein Extraction Kit (Beyotime Biotechnology, Shanghai, China) and quantitated by Pierce™ Rapid Gold BCA Protein Assay Kit (Thermo Scientific, Waltham, MA, USA). Samples were analyzed on the sodium dodecylsulfate (SDS)-polyacrylamide gels electrophoresis (PAGE) and then transferred onto polyvinylidene fluoride (PVDF) membranes (Santa Cruz Biotech, Dallas, TX, USA). 5% bovine serum albumin (BSA; Thermo Scientific, Waltham, MA, USA) was used for blocking, and primary rabbit polyclonal antibodies purchased from Abcam (Abcam, Cambridge, MA, USA) including SOX4 (1:800), E-cadherin (1:800), N-cadherin (1:1200), Snail (1:1000) and Vimentin (1:1000) were applied onto the PVDF membranes at 4 °C for overnight incubation, respectively. The internal reference was a primary rabbit polyclonal antibody against GADPH (1:2500; Abcam, Cambridge, MA, USA). After washing, a horseradish-peroxidase-conjugated against rabbit antibody (1:2000; Abcam, Cambridge, MA, USA) was applied onto the PVDF membranes at room temperature for 1 h. An ECL detection kit (Abcam, Cambridge, MA, USA) was used to detect the results. Image J software (NIH, Bethesda, Maryland, USA) was used to calculated the grey level ratio of target protein to internal reference.

### Cell immunofluorescence

ARPE19 cells at 10^6^ cells/mL were cultured in slices which used for cell immunofluorescence. Cells were firstly fixed with paraformaldehyde (4%, Sigma-Aldrich, St Louis, MS, USA), and then applied with Triton X-100 (0.1%; Sigma-Aldrich, St Louis, MS, USA) for permeabilization, and blocked with BSA (1%; Thermo Scientific, Waltham, MA, USA). After washing, the primary rabbit polyclonal antibodies purchased from Abcam (Abcam, Cambridge, MA, USA) including SOX4 (1:600), E-cadherin (1:800), N-cadherin (1:1100), Snail (1:800) and Vimentin (1:1000) were added on the slides at 4 °C for overnight incubation. A fluorescent goat anti-rabbit polyclonal secondary antibody (1:1200; Abcam, Cambridge, MA, USA) was incubated for 30 min at room temperature. Nuclei were stained with DAPI. A fluorescence microscope (Leica, Wetzlar, Germany) was used to observe the results of protein expression.

### Wound healing assay

Following previously described (Cory, 2011), ARPE19 cells at 10^6^ cells/mL were cultured in 6-well plate. A 10 µL pipette tip was used for scratch at the center of each well and then cells were incubated with serum-free DMEM and high glucose for 72 h. For combination with small interfering RNA (siRNA) transfection, ARPE19 cells were first transfected with siRNA for 24 h, then incubated with high glucose medium for 72 h. The images of each well of 6-well plate were pictured by an inverted microscope (Leica, Wetzlar, Germany) and analyzed through ImageJ software (NIH, Bethesda, Maryland, USA) to calculate the relative distance according to the equation (W_0_–W _t_)/W_0_ ×100%.

### Transwell assay

ARPE19 cells at 10^4^ cells/mL were cultured in the upper chamber with Matrigel-coated membrane (BD Biosciences, Bedford, MA, USA) under serum-free culture medium for 72 h incubation. Using a cotton swab, the non-invading cells on the upper chamber were eliminated. After fixing with paraformaldehyde (4%, Sigma-Aldrich, St Louis, MS, USA), the cells on the lower side of the chamber were incubated with crystal violet. The results were counted in five fields randomly selected by a microscope (Leica, Wetzlar, Germany).

### Cell transfection

The NEAT1 knockdown plasmid (si-NEAT1), SOX4 knockdown plasmid (si-SOX4), negative control (NC), miR-204 mimics and miR-204 inhibitor (20 nM) were synthesized by GenePharma (Shanghai, China). For transfection, ARPE19 cells at 5  × 10^5^ cells/mL were cultured into 12-well plate, Lipofectamine 2000 Transfection Reagent (Thermo Fisher Scientific, Waltham, MA, USA) was used to transfect si-NEAT1, si-SOX4, miR-204 mimics or miR-204 inhibitor into cells. For subsequent experiments, cells were harvested after 72 h transfection.

### Dual luciferase reporter assay

Firstly, the starBase database (http://starbase.sysu.edu.cn/index.php) was used for searching the binding site of miR-204 on NEAT1 and SOX4. Then, wild type or mutant NEAT1 fragment (NEAT1-WT or NEAT1-MUT) and wild type or mutant SOX4 3′UTR (SOX4 3′ UTR-WT or SOX4 3′ UTR-MUT) containing the predicted miR-204 binding site were cloned into the pmirGLO luciferase vector (Promega, Madison, WI, USA), respectively, which was co-transfected with miR-204 mimics or mimics NC into ARPE19 cells for 48 h using Lipofectamine 2000 Transfection Reagent (Thermo Fisher Scientific, Waltham, MA, USA). Luciferase Assay System (Promega, Madison, WI, USA) was used to detect the luciferase activity.

### Diabetic mice model

Xiangya Hospital, Central South University Ethics Committee approved the following animal practice (No. 2019-179). 36 healthy male C57BL/6 mice at 8–10 weeks were purchased from Shanghai SLAC Laboratory Animal Co., Ltd., and maintained in the SPF pathogen-free environment. The 36 mice were divided into 4 groups(control group, diabetes model (DM) group, DM+sh-NC group and DM+sh-NEAT1 group) with 3 mice in each group, and the experiment was repeated 3 times. In DM group, streptozotocin (STZ, Sigma-Aldrich, St Louis, MS, USA) was injected intraperitoneally at a dose of 50 mg/kg once a day for 5 days, while in control group an equal volume of citrate buffer was injected. After ensuring that the glucose concentration of tail vein blood was greater than 150 mg/dl three days post-injection, which referred to a previous study (Furman, 2015). ShRNA were administrated with previous study (Fan et al., 2019), mice in DM+sh-NEAT1 group were injected with 1 µL of shRNA targeting NEAT1 in the vitreous cavity, and mice in DM+sh-NC group were injected with the same amount of non-targeting shRNA. Mice were injected every three weeks. After 12 weeks, all mice were humanely euthanized by intraperitoneal injection of barbiturates greater than 150 mg/kg and eyeballs were collected. Each experimental group report includes all animals, experimental units or data points in the analysis.The retina of eyeball tissue was used for glucose concentration measurement by Glucose Assay Kit (Sigma-Aldrich, St Louis, MS, USA).

### Immunohistochemical staining

For immunohistochemical staining, retinas from different group of diabetic mice were fixed with paraformaldehyde (10%, Sigma-Aldrich, St Louis, MS, USA). After cut serially, slides (5 µm) were boiled in citrate buffer solution (10 mM, pH 6.0) for 20 min of antigen retrieval and then blocked with 0.3% hydrogen peroxide to eliminate the endogenous peroxidase activity. After incubating with a primary rabbit polyclonal antibody against SOX4 (1:600; Abcam, Cambridge, MA, USA), E-cadherin (1:500), N-cadherin (1:800), Snail (1:500) and Vimentin (1:600) at 4 °C for 12 h, the slides were applied with a HRP-labeled goat anti-rabbit secondary antibody (1:1200; Abcam, Cambridge, MA, USA) for 30 min and a DAB detection kit (Abcam, Cambridge, MA, USA) for 10 min. After dehydrated and cleared, the slides were analyzed under a microscopy (Leica, Wetzlar, Germany).

### Statistical analysis

All experiments were conducted at least three independent replications in three times. Presenting as mean ± SD, data were analyzed by Student’s *t* test (for two groups) or one-way analysis of variance (ANOVA)(for three or more than three groups). Differences calculated by GraphPad Prism 6.0 software were considered statistically significant at *p* <0.05.

**Figure 1:**
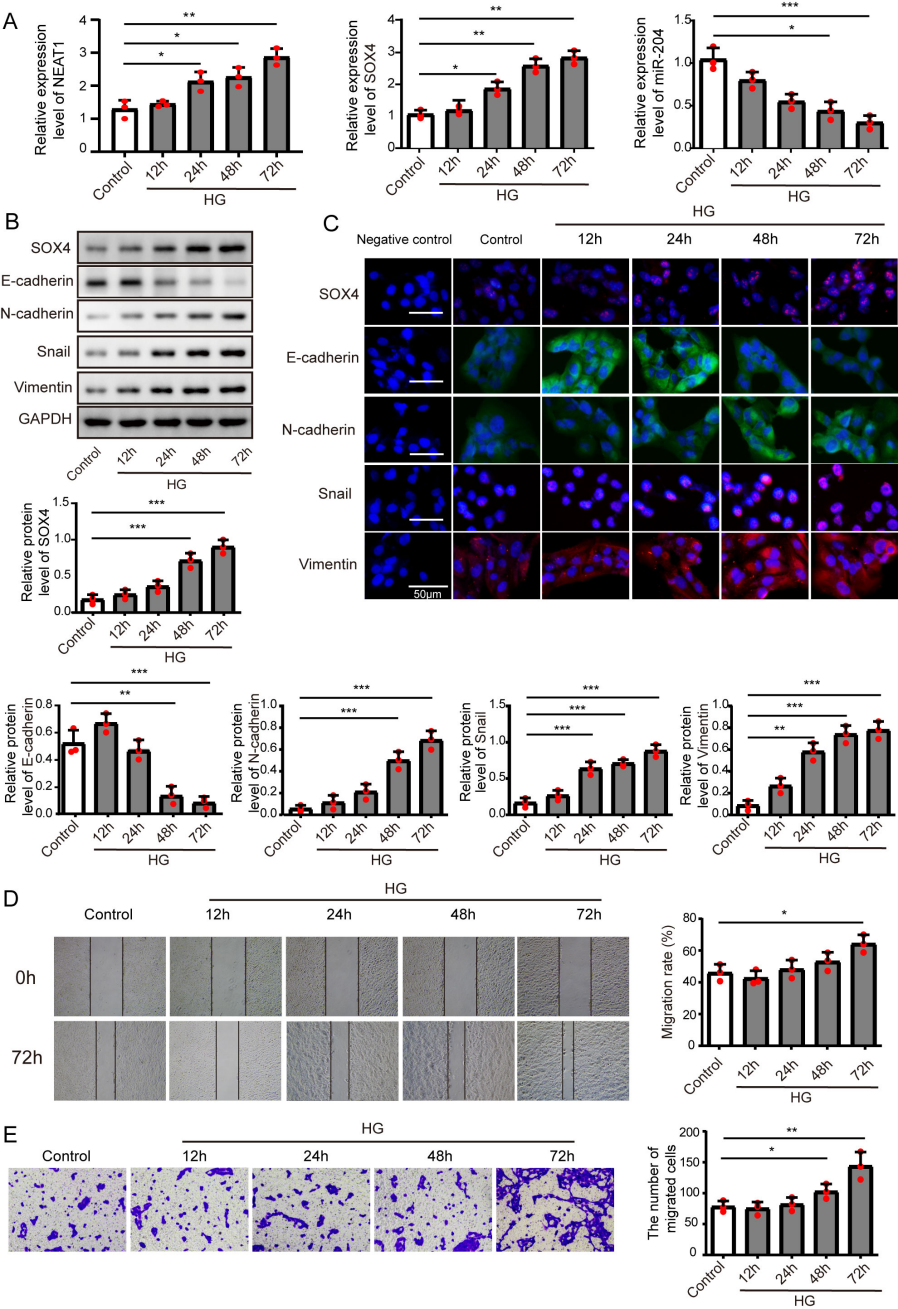
High glucose upregulated NEAT1 and SOX4, and downregulated miR-204. (A) qRT-PCR analysis of NEAT1, SOX4 and miR-204 in ARPE19 cells at 12, 24, 48 and 72 h after high glucose induction. (B–C) SOX4 and EMT-associated proteins such as E-cadherin, N-cadherin, Snail, and Vimentin were examined by western blot (B) and cell immunofluorescence (C) at 12, 24, 48 and 72 h after high glucose induction, Scale bar: 50 µM. D. Cell migration was investigated by wound healing assay, the first row was the scratch size of control and high glucose treatment (12, 24, 48 and 72 h) group at 0 h, which was used as a baseline comparison, while the second row was the scratch size of corresponding groups at 72 h, which could be used to evaluate the treatment effects against high glucose by comparing with the first row. E. invasion ability was investigated by transwell assay E at 12, 24, 48 and 72 h after high glucose induction. Data were presented as the mean ± SD of three separated experiments. ^∗^*p* < 0.05, ^∗∗^*p* < 0.01, ^∗∗∗^*p* < 0.001.

## Results

### High glucose upregulated NEAT1 and SOX4, and downregulated miR-204

ARPE19 cells were treated without or with high glucose for 12, 24, 48 and 72 h, and subjected to qRT-PCR assay to detect the expression of NEAT1, miR-204 and SOX4 (Fig. 1A), with the increase of induction time in the high glucose group, NEAT1 and SOX4 were gradually up-expressed, and miR-204 was gradually down-expressed. Also, the protein level of SOX4 and the EMT related proteins such as E-cadherin, N-cadherin, Snail and Vimentin were evaluated by western blot and cell immunofluorescence. As shown in Figs. 1B and 1C, the protein level of SOX4 also displayed a time-dependent increase, the level of E-cadherin was reduced gradually and the concentrations of N-cadherin, Snail and Vimentin were increased, indicating high glucose-induced EMT progression. In Fig. 1D, the first row was the scratch size of control and high glucose treatment (12, 24, 48 and 72 h) group at 0 h, which was used as a baseline comparison, while the second row was the scratch size of corresponding groups at 72 h. The wound healing assay showed that high glucose promoted the migration of ARPE19 cells. The results of transwell assay in Fig. 1E also revealed that high glucose induced time-dependent migration of ARPE19 cells. Therefore, high glucose could regulate NEAT1, SOX4 and miR-204, thus consequently accelerated the EMT progression.

### Knockdown of NEAT1 inhibited high glucose-induced EMT

**Figure 2:**
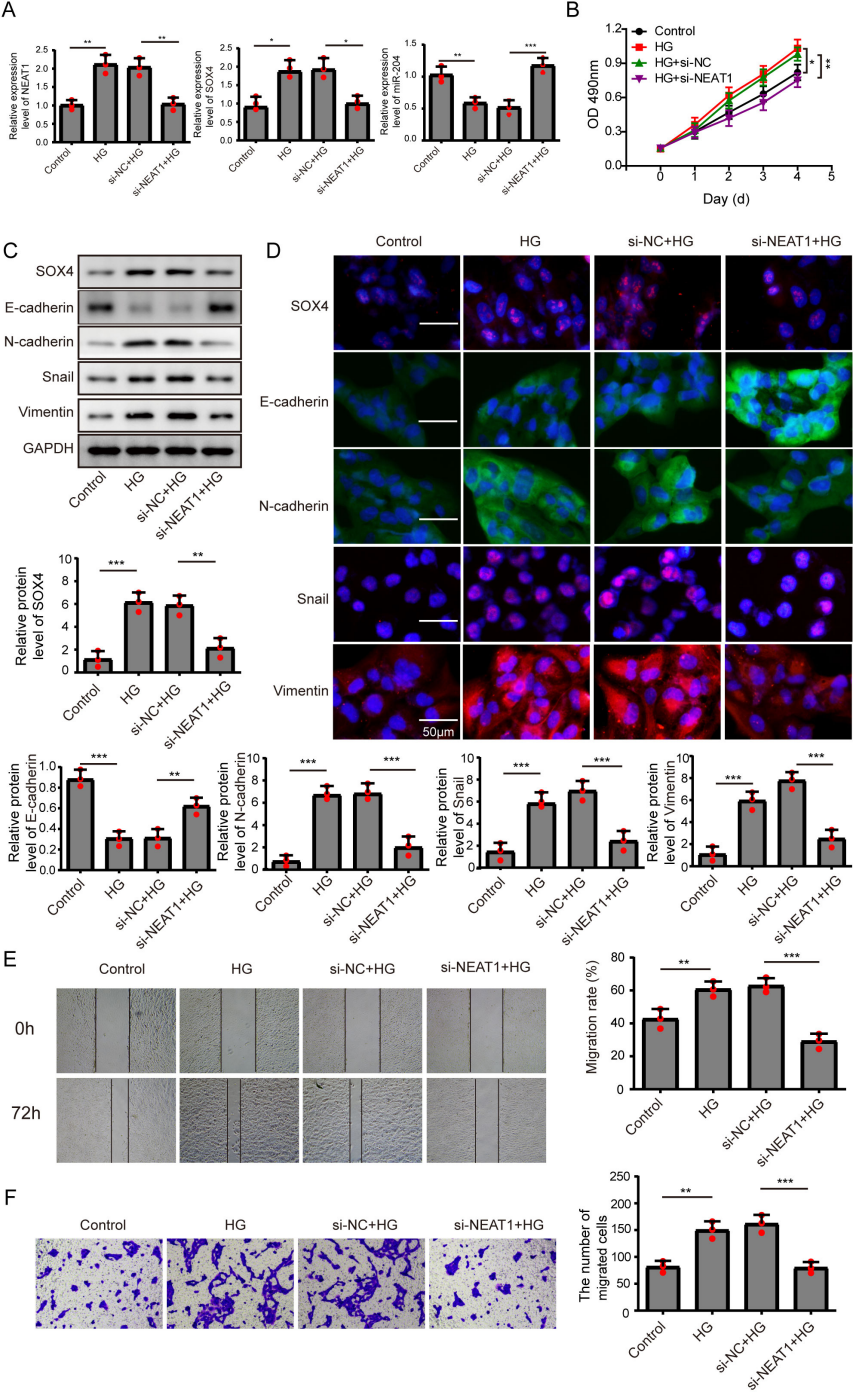
Knockdown of NEAT1 inhibited high glucose-induced EMT. (A) ARPE19 cells was first transfected with si-NEAT1, then the expression level of NEAT1, SOX4 and miR-204 after 72 h high glucose induction were analyzed by qRT-PCR. (B) Cell viability were verified by MTT assay in experiments described in A. (C–D). SOX4 and EMT-associated proteins such as E-cadherin, N-cadherin, Snail, and Vimentin were examined by western blot (C) and cell immunofluorescence (D) after 72 h high glucose induction and 72 h si-NEAT1 transfection. Scale bar: 50 µM. (E–F). Cell migration and invasion ability was investigated by wound healing assay (E) and cell transwell assay (F). Data were presented as the mean ± SD of three separated experiments. ^∗^*p* < 0.05, ^∗∗^*p* < 0.01, ^∗∗∗^*p* < 0.001.

In order to detect the NEAT1-mediated effects and explore the mechanism of NEAT1 in EMT induced by high glucose, ARPE19 cells was transfected with small interfering RNA (siRNA) of NEAT1. After si-NEAT1 transfection, the high expression level of NEAT1 induced by high glucose was reduced, indicating a successful transfection. The inhibition of miR-204 and increasement of SOX4 induced by high glucose could be reversed by si-NEAT1 transfection (Fig. 2A). Because the capacity of cell proliferation was one of the most basic characteristics of cell function, we conducted MTT and BrdU assay to reflect the effect of high glucose on cell viability. The results showed that knockdown of NEAT1 reversed the effect of high glucose on promoting cell viability and cell proliferation (Fig. 2B, Supplement S1A). Western blot and cell immunofluorescence assay displayed that although SOX4, N-cadherin, Snail and Vimentin were up-regulated in high glucose group, an opposite expression trend of those proteins was observed in high glucose + si-NEAT1 group (Figs. 2C, 2D), revealing that knockdown of NEAT1 may reversed the high glucose-induced EMT progression. In Fig. 2E, the first row was the scratch size of each group at 0 h, while the second row was the scratch size of corresponding groups at 72 h after high glucose treatment. The results from Figs. 2E and 2F found out that high glucose led to cell migration and knockdown of NEAT1 could reversed the above situation.

**Figure 3:**
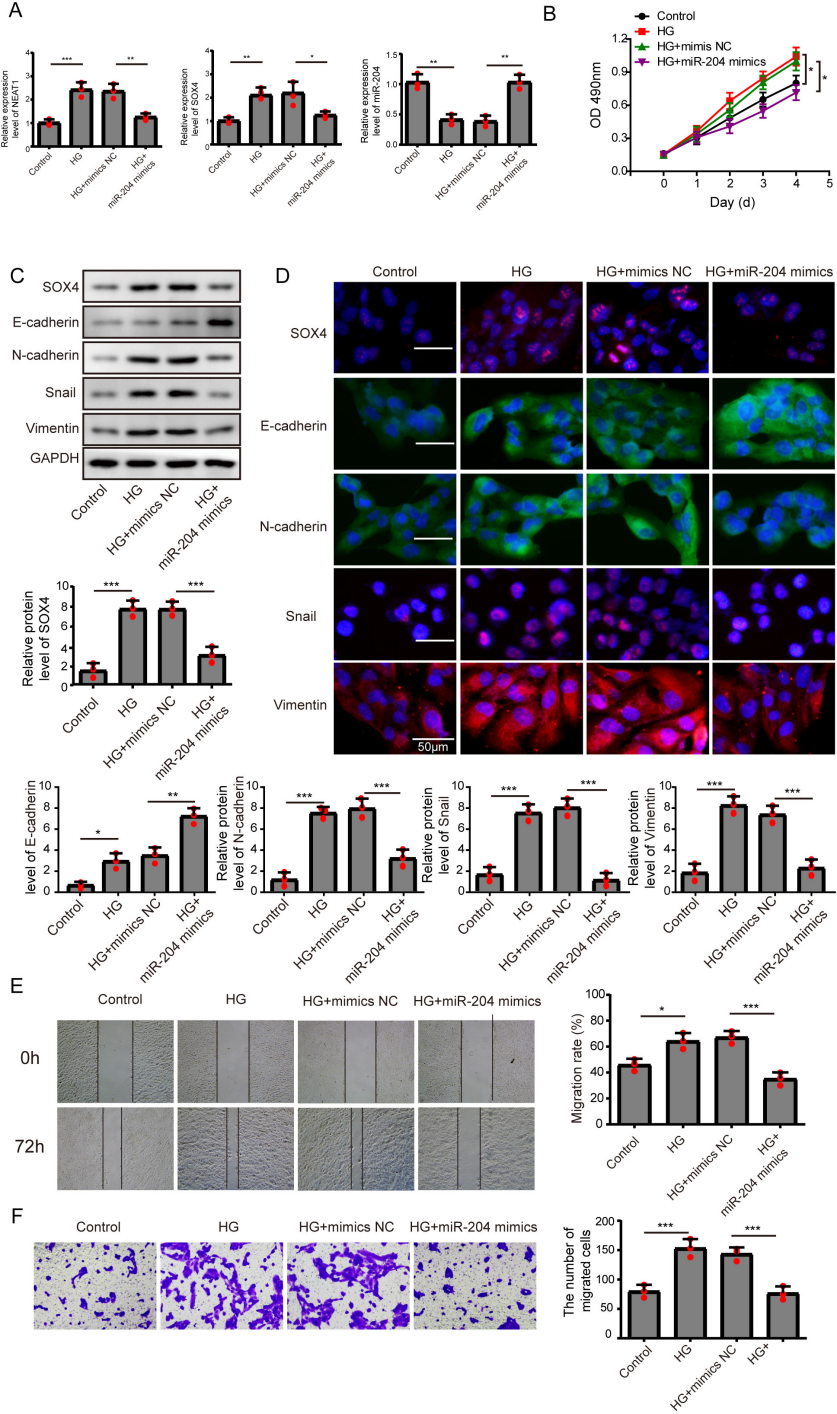
Overexpression of miR-204 inhibited EMT progression induced by high glucose. (A) NEAT1, miR-204 and SOX4 in ARPE19 cells after 72 h high glucose induction and 72 h miR-204 mimic transfection was analyzed by qRT-PCR. (B) Cell viability was verified by MTT assay after 72 h high glucose induction and 72 h miR-204 mimic transfection. (C–D). SOX4 and EMT-associated proteins such as E-cadherin, N-cadherin, Snail, and Vimentin were examined by western blot (C) and cell immunofluorescence (D) after 72 h high glucose induction and 72 h miR-204 mimic transfection. Scale bar: 50 µM. (E–F). Cell migration and invasion ability was investigated by wound healing assay (E) and cell transwell assay (F) after 72 h high glucose induction and 72 h miR-204 mimic transfection. Data were presented as the mean ± SD of three separated experiments. ^∗^*p* < 0.05, ^∗∗^*p* < 0.01, ^∗∗∗^*p* < 0.001.

### Overexpression of miR-204 inhibited EMT progression induced by high glucose

In order to detect the miR-204-mediated effects on EMT progression induced by high glucose, ARPE19 cells transfected with miR-204 mimics were used for experiments after 72 h transfection. The expressed concentrations of NEAT1, miR-204 and SOX4 were firstly examined by qRT-PCR analysis. The lower concentration of miR-204 induced by high glucose was increased after miR-204 mimics transfection. Also, high glucose increased the expressions of NEAT1 and SOX4, which could be reversed by miR-204 mimic transfection (Fig. 3A). What’s more, the results of MTT assay and BrdU assay revealed that miR-204 mimics inhibited cell viability promoted by high glucose (Fig. 3B, Supplement S1B). Western blot and cell immunofluorescence assay displayed the expressions of SOX4, N-cadherin, Snail and Vimentin were up-regulated and the expression of E-cadherin was reduced under high glucose condition, and an opposite expression trend of those proteins was observed by miR-204 mimics transfection (Figs. 3C and 3D), revealing that overexpression of miR-204 may reversed the high glucose-induced EMT progression. In Fig. 3E, the treatment effects against high glucose were evaluated comparing the first row at 0 h with the second row at 72 h. High glucose accelerated migration and invasion of ARPE19 cells, which was reversed by miR-204 overexpression (Figs. 3E, 3F).

### NEAT1 could function as a competing endogenous RNA(ceRNA) to affect SOX4 through sponging miR-204

**Figure 4:**
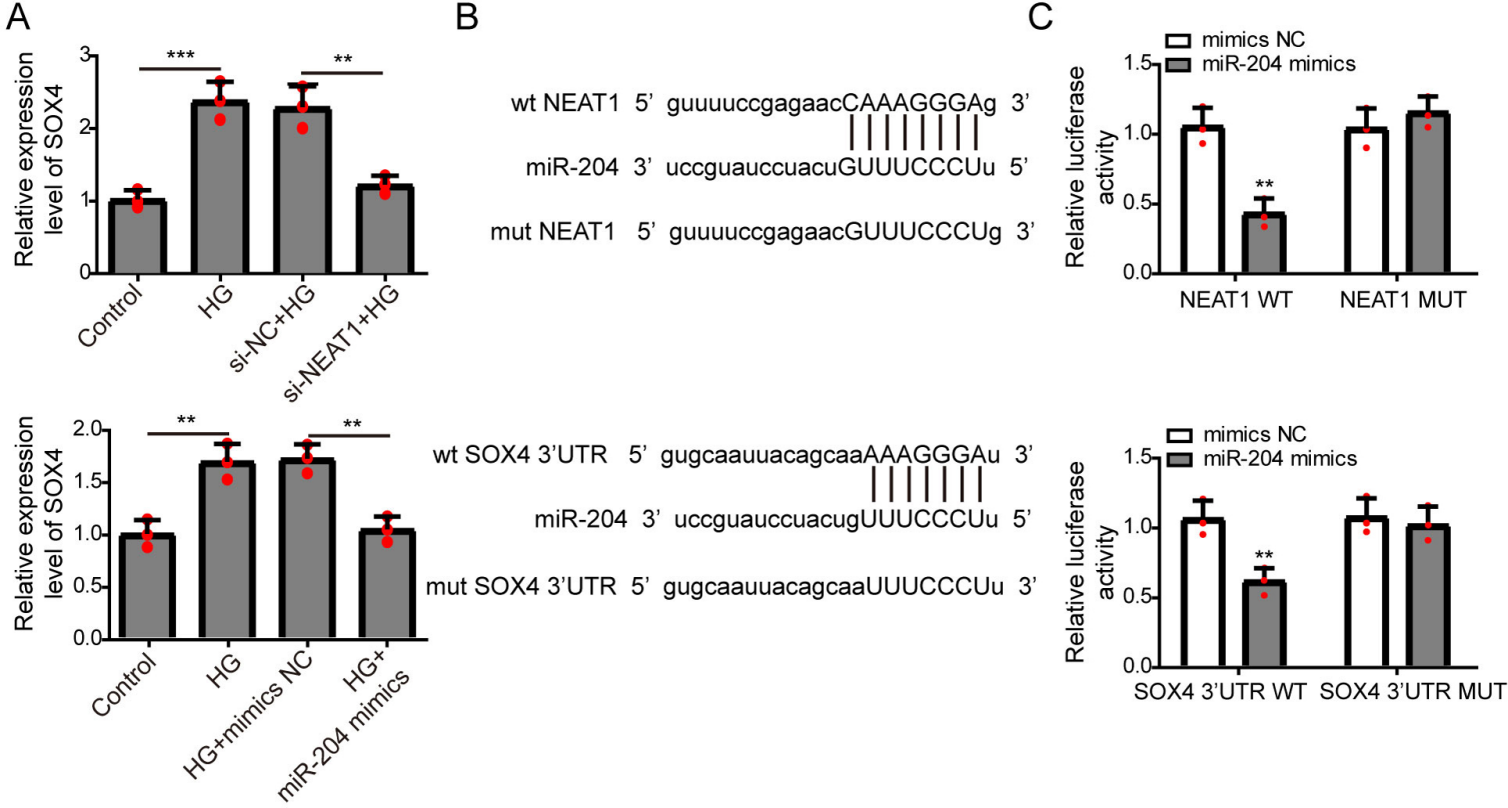
NEAT1 could function as a ceRNA to affect SOX4 through sponging miR-204. (A) ARPE19 cells were first transfected with si-NEAT1 or miR-204 mimics, then the expression level of SOX4 after 72 h high glucose induction were analyzed by qRT-PCR. (B) Putative binding site between miR-204 and NEAT1 or SOX4. (C) The luciferase activity was detected by a dual luciferase reporter system.

The expression of SOX4 mRNA in ARPE19 cells after 72 h si-NEAT1 or miR-204 mimics transfection was firstly evaluated by qRT-PCR analysis. NEAT1 knockdown or miR-204 overexpression could both inhibit the increased expression of SOX4 induced by high glucose (Fig. 4A). Then, the potential binding sites of NEAT1 and SOX4 for miR-204 were predicted using bioinformatics analysis, demonstrating that NEAT1 and the 3′UTR region of SOX4 had a binding site for miR-204 (Fig. 4B). The relationship between NEAT1, SOX4 and miR-204 was verified by dual luciferase reporter assay. The relationship between NEAT1, SOX4 and miR-204 was verified by dual luciferase reporter assay, showing that the luciferase reporter activities were lower in groups of ARPE19 cells co-transfecting with pmirGLO-NEAT1-WT vector or pmirGLO-SOX4 3′UTR-WT and miR-204 mimic. while, the luciferase activities of cells co-transfecting with pmirGLO-NEAT1-MUT vector or pmirGLO-SOX4 3′UTR-MUT vector and and miR-204 mimic or negative control had no change (Fig. 4C).

**Figure 5:**
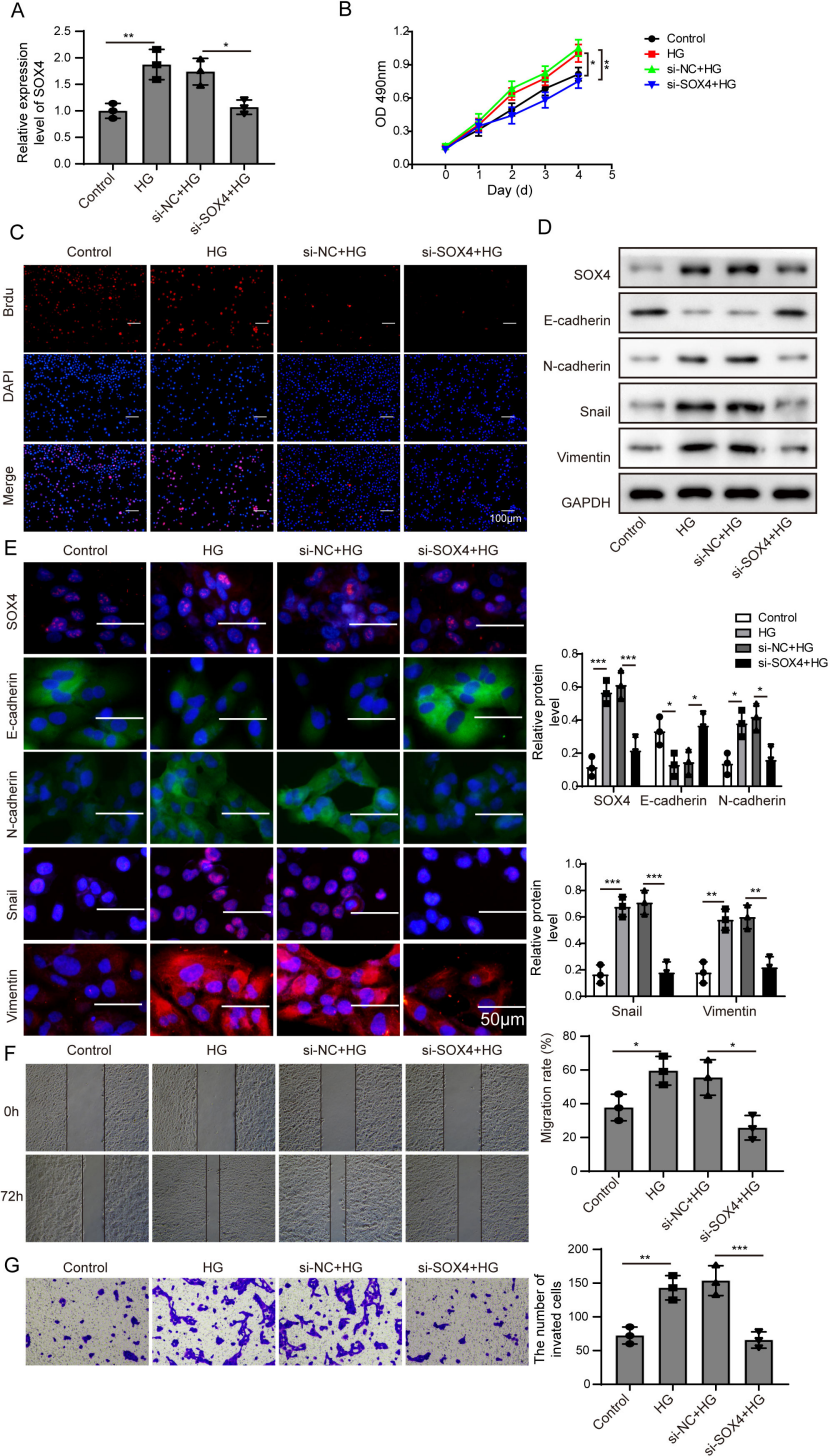
NEAT1 could function as a ceRNA to affect SOX4 through sponging miR-204. (A) SOX4 in ARPE19 cells after 72 h high glucose induction and 72 h si-SOX4 transfection was analyzed by qRT-PCR. (B–C). Cell viability and cell proliferation were verified by MTT assay(B) and BrdU assay (C) respectively after 72 h high glucose induction and 72 h si-SOX4 transfection. (D–E). SOX4 and EMT-associated proteins such as E-cadherin, N-cadherin, Snail, and Vimentin were examined by western blot (D) and cell immunofluorescence (E) after 72 h high glucose induction and 72 h si-SOX4 transfection. Scale bar: 50 µM. (F–G) Cell migration and invasion ability was investigated by wound healing assay (F) and cell transwell assay (G) after 72 h high glucose induction and 72 h si-SOX4 transfection. Data were presented as the mean ± SD of three separated experiments. ^∗^*p* < 0.05, ^∗∗^*p* < 0.01, ^∗∗∗^*p* < 0.001.

Then, SOX4 was blocked to verify its effect on MTT. Firstly, the mRNA level of SOX4 was decreased after si-SOX4 transfection (Fig. 5A). The results of MTT assay and BrdU assay verified that after knocking down SOX4, cell proliferation activity was inhibited (Figs. 5B, 5C) and cell immunofluorescence assay (Fig. 5E) showed that comparing to that of high glucose group, the expression of SOX4, N-cadherin, Snail and Vimentin were decreased except E-cadherin, , and inhibition of SOX4 reversed the progression of high glucose-induced EMT. In Fig. 5F, the treatment effects against high glucose were evaluated comparing the first row at 0 h with the second row at 72 h. Knockdown of SOX4 could inhibit the high glucose-induced migration and invasion of ARPE19 cells (Figs. 5F, 5G). Therefore, it is suggested that NEAT1 could function as a ceRNA to affect SOX4 through sponging miR-204.

**Figure 6:**
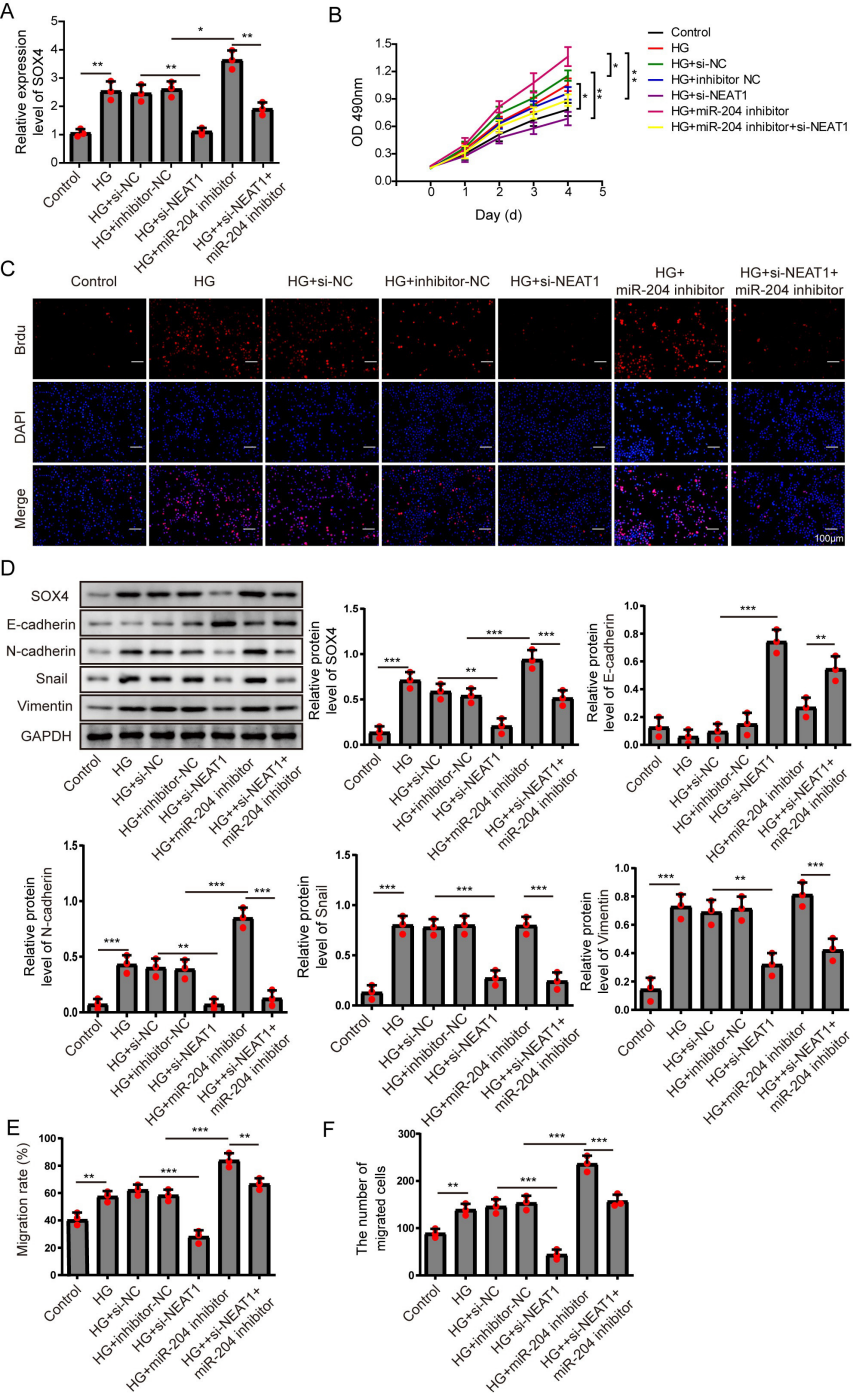
NEAT1 regulated high glucose-induced EMT through miR-204/SOX4 pathway. (A) SOX4 in ARPE19 cells after 72 h high glucose induction and 72 h si-NEAT1 and miR-204 inhibitor co-transfection was analyzed by qRT-PCR. (B–C). Cell viability and cell proliferation were verified by MTT assay (B) and BrdU assay (C) respectively after 72 h high glucose induction and 72 h si-NEAT1 and miR-204 inhibitor co-transfection. (D) SOX4 and EMT-associated proteins such as E-cadherin, N-cadherin, Snail, and Vimentin were examined by western blot after 72 h high glucose induction and 72 h si-NEAT1 and miR-204 inhibitor co-transfection. (E–F) Cell migration and invasion ability was investigated by wound healing assay and cell transwell assay after 72 h high glucose induction and 72 h si-NEAT1 and miR-204 inhibitor co-transfection. Data were presented as the mean ± SD of three separated experiments. ^∗^*p* < 0.05, ^∗∗^*p* < 0.01, ^∗∗∗^*p* < 0.001.

### NEAT1 regulated high glucose-induced EMT through miR-204/SOX4 pathway

To investigate the mechanism of NEAT1 on EMT progression mediated by high glucose, NEAT1 siRNA and miR-204 inhibitor were co-transfected into ARPE19 cells. In Fig. 6A, the mRNA expression of SOX4 was elevated in high glucose group and si-NEAT1 inhibited the higher level of SOX4 mediated by high glucose. Then with the treatment of miR-204 inhibitor alone, the increased expression of SOX4 induced by high glucose was aggravated. In miR-204 inhibitor and si-NEAT1 co-transfected cells the expression of SOX4 was slightly decreased compared with the control group. The changes of cell viability from MTT assay were consistent with the trend of cell proliferation tested by BrdU assay (Figs. 6B, 6C). The cell viability and cell proliferation was significant promoted in high glucose group which could be inhibited by si-NEAT1. Then with the treatment of miR-204 inhibitor alone, the promoted cell viability and cell proliferation by high glucose was aggravated. However, miR-204 inhibitor could reverse inhibitory effect of si-NEAT1 on cell viability and cell proliferation in co-transfected cells. The changes of SOX4 and EMT-associated proteins including E-cadherin, N-cadherin, Snail and Vimentin in ARPE19 cells co-transfecting with si-NEAT1 and miR-204 inhibitor were evaluated. High glucose promoted the expression of SOX4, N-cadherin, Snail and Vimentin, and inhibited E-cadherin expression, which was more prominently after miR-204 inhibitor transfection, while si-NEAT1 inhibited SOX4, N-cadherin, Snail, Vimentin, and promoted E-cadherin expression. In addition, miR-204 inhibitor could reverse the regulated effects of si-NEAT1 on SOX4 and EMT-related proteins (Fig. 6D). Correspondingly, high glucose accelerated cell migration and invasion, and si-NEAT1 inhibited high glucose-promoted cell migration and invasion, and the inhibitory effect of si-NEAT1 were ameliorated after miR-204 inhibitor transfection (Figs. 6E, 6F). All the results demonstrated that NEAT1 regulated high glucose-induced EMT of ARPE19 cells through miR-204/SOX4 pathway.

**Figure 7:**
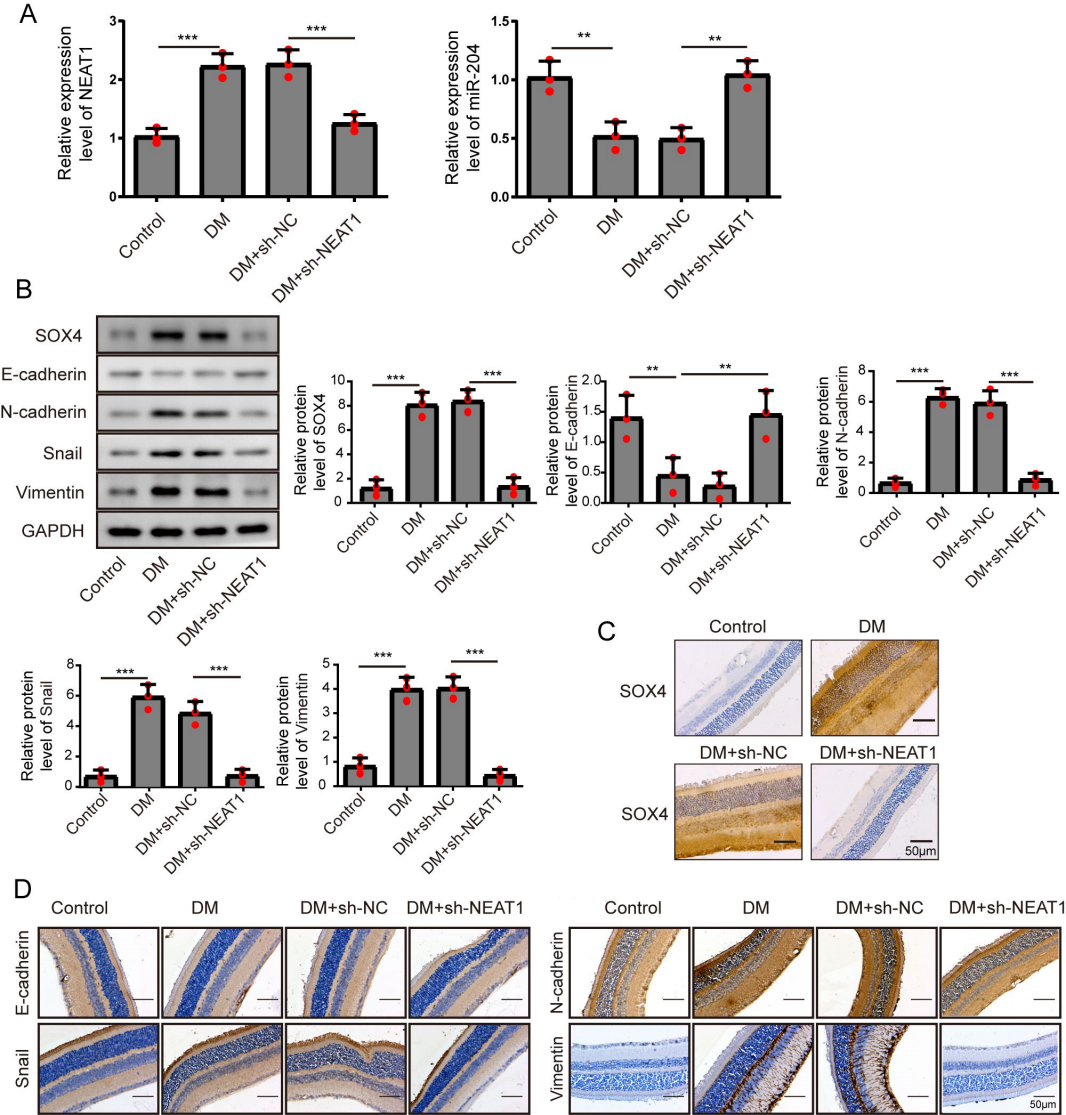
NEAT1 regulated EMT and miR-204/ SOX4 pathway in the retina of diabetic mice. (A) NEAT1 and miR-204 was analyzed by qRT-PCR. (B) SOX4 and EMT-associated proteins such as E-cadherin, N-cadherin, Snail and Vimentin were examined by western blot. (C) SOX4 in the retina of diabetic mice was assessed by immunohistochemical staining assay. Scale bar: 50 µM. (D) E-cadherin, N-cadherin, Snail and Vimentin in the retina of diabetic mice were assessed by immunohistochemical staining assay. Scale bar: 50 µM. Data were presented as the mean ± SD of three separated experiments. ^∗^*p* < 0.05, ^∗∗^*p* < 0.01, ^∗∗∗^*p* < 0.001.

**Figure 8:**
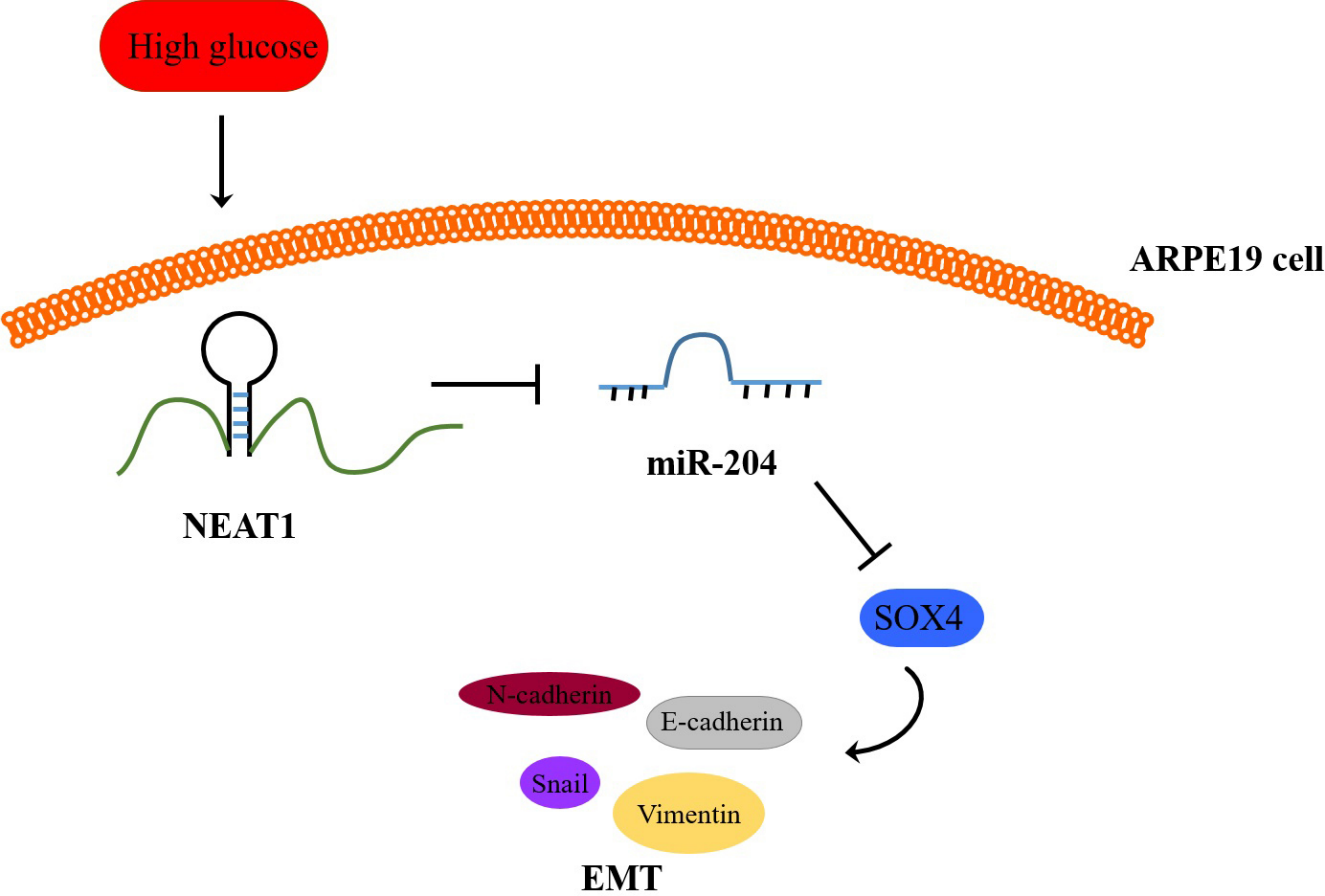
Schematic representation of conclusions drawn from this study showing NEAT1 regulated the development of EMT in DR through the miR-204/SOX4 pathway.

### NEAT1 regulated EMT and miR-204/ SOX4 pathway in the retina of diabetic mice

To investigate the effect of downregulation of NEAT1 in the retina of diabetic mice, we constructed a diabetic model of mice and the retinas were isolated from diabetic mice. NEAT1 was up-expressed and miR-204 was down-expressed in retina of mice in DM group. After treatment with NEAT1 shRNA, NEAT1 was decreased and miR-204 was increased (Fig. 7A). In Fig. 7B, the protein levels of SOX4, N-cadherin, Snail and Vimentin were upregulated and E-cadherin was down-regulated in DM group, which was reversed by the NEAT1 shRNA treatment. The distribution of SOX4 in the retina of diabetic mice was increased, and in DM + sh-NEAT1 group the increased distribution of SOX4 was ameliorated (Fig. 7C). Also, the distributions of N-cadherin, Snail, Vimentin and E-cadherin in Fig. 7D were consistent with the results of western blot. According to these results, NEAT1 regulated high glucose-induced EMT and miR-204/SOX4 pathway in the retina of diabetic mice. As a schematic diagram of our study, Fig. 8 concluded that NEAT1 could regulate the progression of EMT in DR through miR-204/SOX4 pathway.

## Discussion

As a common vascular complication of diabetes, DR is a familiar reason for blindness (Group & Group, 2010). About 23% of diabetic patients in China would develop into progressive disease (Xu et al., 2013) with significant pathological change of EMT. Fibroblast-like transformation of retinal pigment epithelial (RPE) cells is a pathological feature that may cause blindness. In this study, the relationship between NEAT1, miR-204 and SOX4 in EMT progression of DR was investigated. Firstly, the expression trend of lncRNA NEAT1, miR-204 and SOX4 in ARPE19 cells were discovered in high glucose condition and high glucose promoted EMT progression of ARPE19 cells. Then, by transfecting si-NEAT1, miR-204 mimics and miR-204 inhibitor, we revealed that NEAT1 knockdown or miR-204 overexpression suppressed the high glucose-mediated EMT progression in ARPE19 cells. Also, we verified the mechanism of NEAT1 mediated SOX4 to regulate EMT induced by high glucose through targeting miR-204 in ARPE19 cells. The cell used in this study was ARPE-19 cell instead of primary hRPE cells. Primary hRPE cells were difficult to culture, and with a limited number of passages, while ARPE-19 has structural and functional properties characteristic of hRPE cells and is suitable for *in vitro* studies of primary hRPE cells physiology. The relationship between other microRNAs such as miR-302d/miR-93 and EMT has been studied on ARPE-19 cell (Fuchs et al., 2020).

In the human genome, less than 2% of the gene sequences are involved in encoding proteins, and more than 98% of the gene sequences are non-coding sequences (Balakirev & Ayala, 2003), and its transcribed fragments are non-coding RNAs. Among them, those with a length of more than 200 nucleotides are lncRNAs (Guttman et al., 2009). Researchers have found that lncRNA was widely involved in many important biological functions in the body (Wapinski & Chang, 2011), and Moran find that that lncRNA was closely related with type 2 diabetes (Morán et al., 2012). As an lncRNA with a length of about 3.2 kb, NEAT1 is important in many tumors by affecting different signaling pathways (Wu et al., 2019; Xiong et al., 2018; Zhang et al., 2017). It is reported that NEAT1 was increased dramatically in ischaemia-reperfused (I/R) treated diabetic myocardial tissues (Ma et al., 2018). In our research, we investigated the regulated effect of NEAT1 in DR and the expression of NEAT1 was enhanced by high glucose *in vitro* and under diabetic conditions *in vivo*. In addition, increasing studies have clearly shown that lncRNA should be considered as a fundamental part of the ceRNA network, because lncRNA can act as miRNA sponges to regulate the protein-coding gene expression. However, it is still not clear how lncRNA-mediated ceRNAs function in DR. It was reported that there was interaction between NEAT1 and miR-204 in breast cancer (Müller et al., 2019). Our study was firstly confirmed the negative relationship of between NEAT1 and miR-204 in ARPE19 cells. NEAT1 knockdown or miR-204 overexpression could inhibit the high glucose-induced cell viability and EMT on ARPE19 cells. MiR-204 was reported to inhibit cell viability of spiral ganglion neurons (SGNs) by targeting transmembrane protease serine3 (TMPRSS3) (Li et al., 2014). What’s more, we demonstrated that by injecting with shRNA targeting NEAT1, EMT progression in the retina of diabetic mice was inhibited. Epithelial marker E-cadherin and mesenchymal markers including Vimentin and N-cadherin were normally investigated to estimate the degree of EMT (Ji et al., 2013; Liu, Cao & Zhao, 2015; Xu et al., 2014).

As a member of the C subfamily of the SOX family, SOX4 is involved in embryonic development and regulation of cell differentiation. In many cancers, abnormal expression of the key transcription factor SOX4 can induce EMT and accelerate cell proliferation (Li et al., 2015; Li et al., 2016; Parvani & Schiemann, 2013). Also, up-expression of SOX4 in diabetic islets was closely related to the reduced insulin secretion and diabetes development (Collins et al., 2016), indicating that high expression of SOX4 gene was correlated positively with the risk of diabetes. In our study, SOX4 was increased in high glucose condition *in vitro* and under diabetic conditions *in vivo*. By co-transfecting with si-NEAT1 and miR-204 inhibitor into ARPE19 cells, it was demonstrated that NEAT1 could mediate miR-204/SOX4 pathway to regulate high glucose-induced EMT. Also, starbase database and dual luciferase assay revealed the targeting site between miR-204 and NEAT1 or SOX4. What’s more, in diabetic mice treated with NEAT1 shRNA, the inhibition of NEAT1 could greatly reduce the expression of SOX4 and therefore relieve EMT progression.

## Conclusions

In conclusion, this research demonstrated that NEAT1 could mediate SOX4 to regulate EMT of DR by targeting miR-204, which provides a new acquaintance and potentially useful therapeutic target of DR.

##  Supplemental Information

10.7717/peerj.11817/supp-1 Supplemental Information 1 Figure S1 A. ARPE19 cells was first transfected with si-NEAT1, then cell proliferation were verified by BrdU assay after 72 h high glucose induction. B. BrdU assay after 72 h high glucose induction and 72 h miR-204 mimic transfection.

10.7717/peerj.11817/supp-2 Supplemental Information 2 Uncropped blots of Fig. 1C

10.7717/peerj.11817/supp-3 Supplemental Information 3 Uncropped blots of Fig. 2C

10.7717/peerj.11817/supp-4 Supplemental Information 4 Uncropped blots of Fig. 3C

10.7717/peerj.11817/supp-5 Supplemental Information 5 Uncropped blots of Fig. 5C

10.7717/peerj.11817/supp-6 Supplemental Information 6 Uncropped blots of Fig. 6B

10.7717/peerj.11817/supp-7 Supplemental Information 7 Author checklist

10.7717/peerj.11817/supp-8 Supplemental Information 8 Raw numeric data

## Additional Information and Declarations

### Data Availability

The following information was supplied regarding data availability: The raw data and western blots are available in the Supplemental Files.
