# Supplementary figures and images for: LncRNA NEAT1 regulated diabetic retinal epithelial-mesenchymal transition through regulating miR-204/SOX4 axis
Source: PeerJ. 2021 Jul 23;9:e11817. doi: 10.7717/peerj.11817 (PMC8312494; doi:10.7717/peerj.11817)

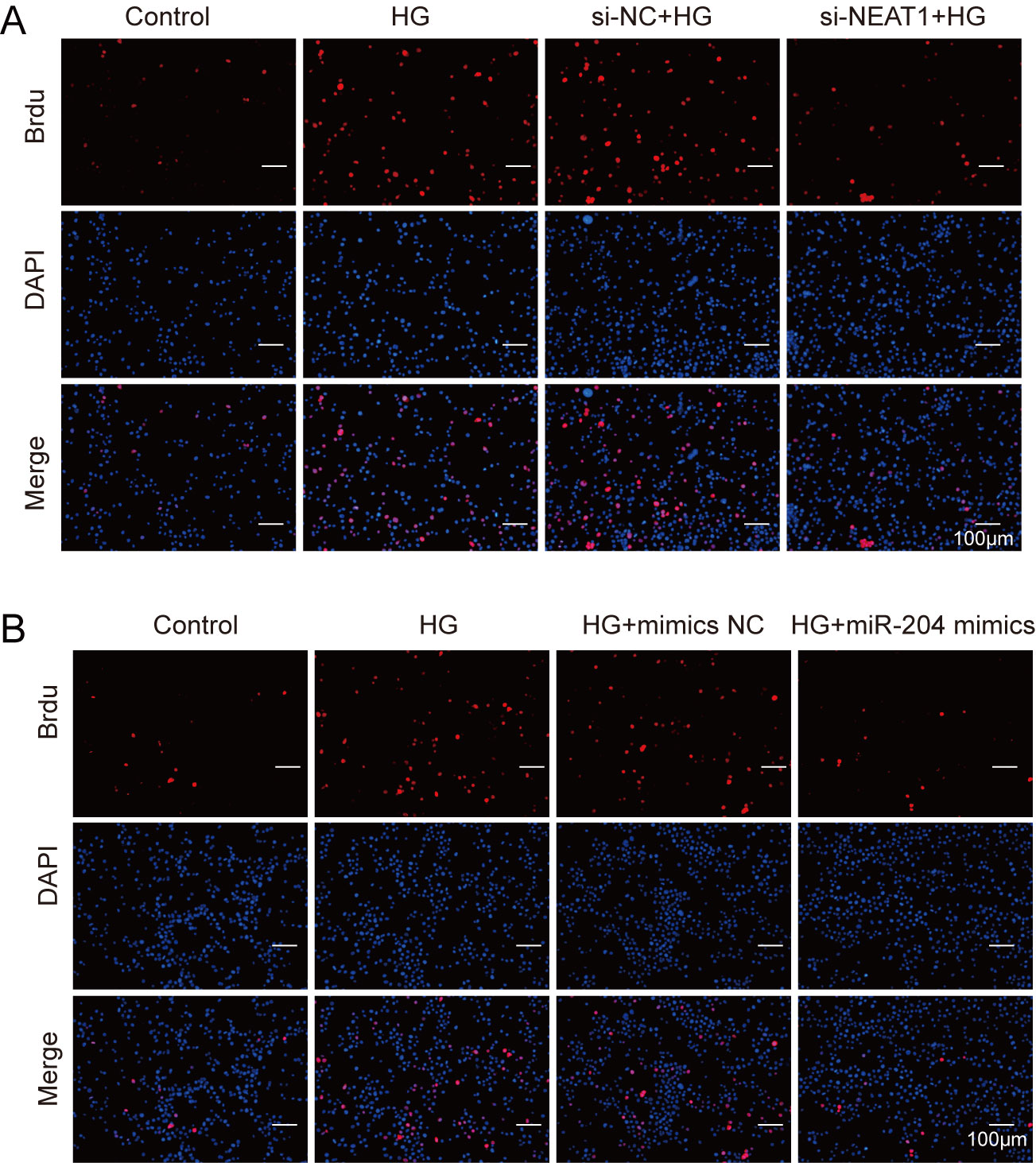

Supplement: Supplemental Information 1 — A. ARPE19 cells was first transfected with si-NEAT1, then cell proliferation were verified by BrdU assay after 72 h high glucose induction. B. BrdU assay after 72 h high glucose induction and 72 h miR-204 mimic transfection. [file peerj-09-11817-s001.jpg]

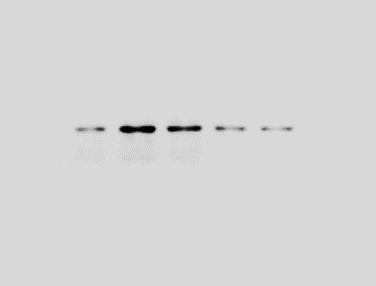

Supplement: Supplemental Information 2 [file peerj-09-11817-s002.zip › figure 1 western blot raw data/E-cadherin.jpg]

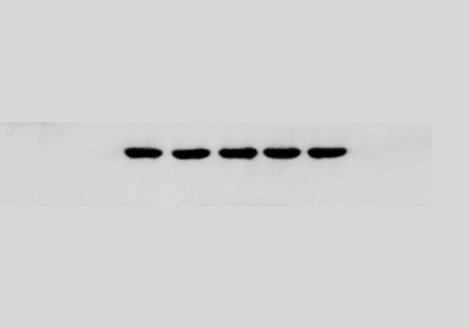

Supplement: Supplemental Information 2 [file peerj-09-11817-s002.zip › figure 1 western blot raw data/GAPDH.jpg]

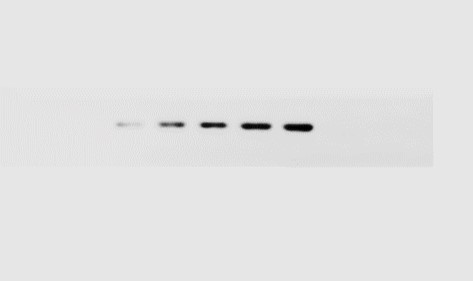

Supplement: Supplemental Information 2 [file peerj-09-11817-s002.zip › figure 1 western blot raw data/N-cadherin.jpg]

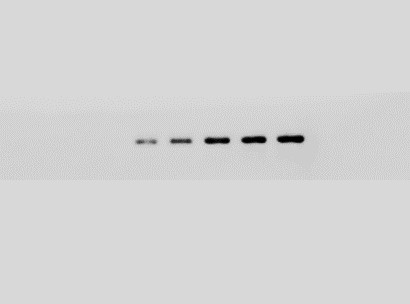

Supplement: Supplemental Information 2 [file peerj-09-11817-s002.zip › figure 1 western blot raw data/Snail.jpg]

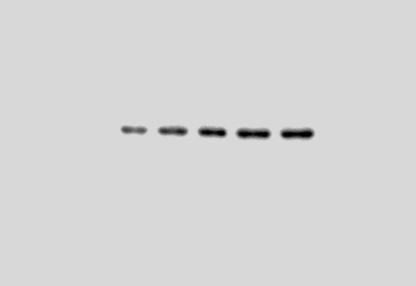

Supplement: Supplemental Information 2 [file peerj-09-11817-s002.zip › figure 1 western blot raw data/Vimentin.jpg]

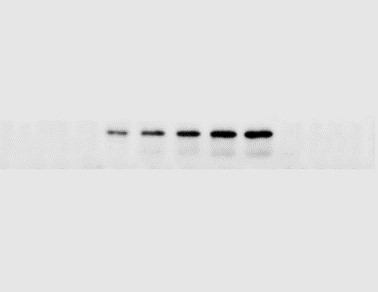

Supplement: Supplemental Information 2 [file peerj-09-11817-s002.zip › figure 1 western blot raw data/sox4.jpg]

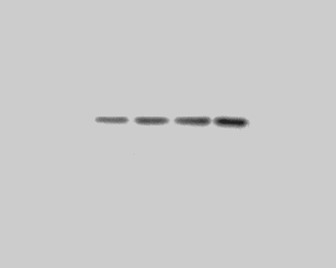

Supplement: Supplemental Information 3 [file peerj-09-11817-s003.zip › figure 2 western blot raw data/E-cadherin.jpg]

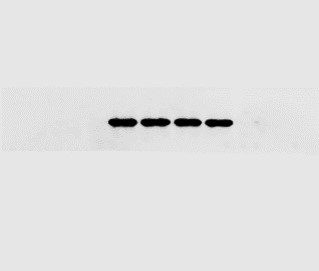

Supplement: Supplemental Information 3 [file peerj-09-11817-s003.zip › figure 2 western blot raw data/GAPDH.jpg]

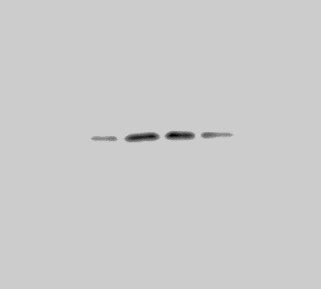

Supplement: Supplemental Information 3 [file peerj-09-11817-s003.zip › figure 2 western blot raw data/N-cadherin.jpg]

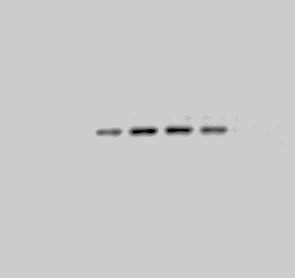

Supplement: Supplemental Information 3 [file peerj-09-11817-s003.zip › figure 2 western blot raw data/SOX4.jpg]

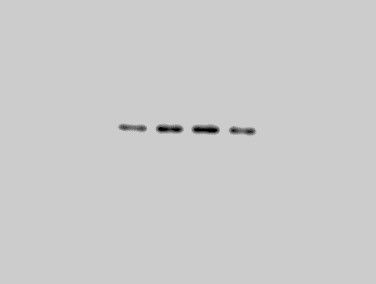

Supplement: Supplemental Information 3 [file peerj-09-11817-s003.zip › figure 2 western blot raw data/Snail.jpg]

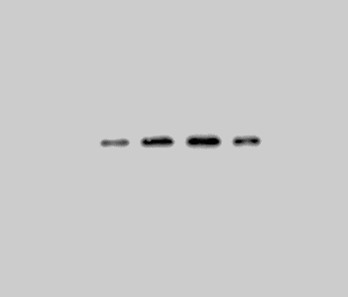

Supplement: Supplemental Information 3 [file peerj-09-11817-s003.zip › figure 2 western blot raw data/Vimentin.jpg]

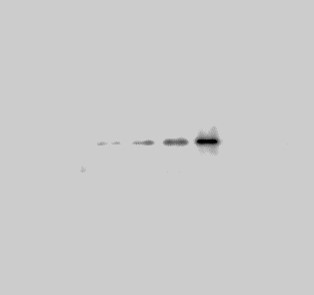

Supplement: Supplemental Information 4 [file peerj-09-11817-s004.zip › figure 3 western blot raw data/E-cadherin.jpg]

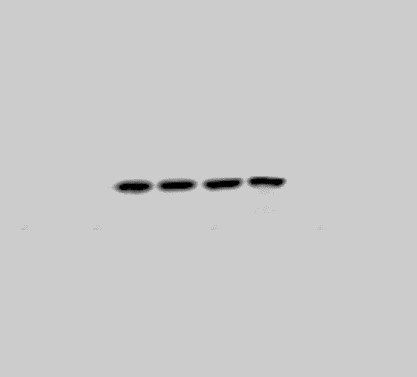

Supplement: Supplemental Information 4 [file peerj-09-11817-s004.zip › figure 3 western blot raw data/GAPDH.jpg]

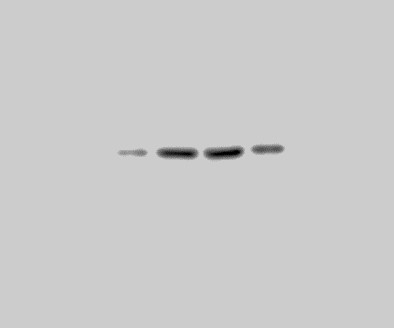

Supplement: Supplemental Information 4 [file peerj-09-11817-s004.zip › figure 3 western blot raw data/N-cadherin.jpg]

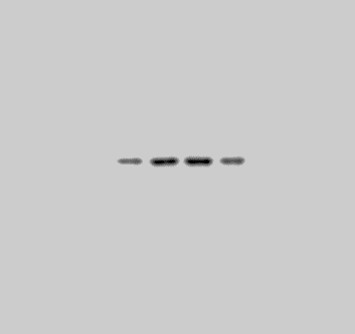

Supplement: Supplemental Information 4 [file peerj-09-11817-s004.zip › figure 3 western blot raw data/SOX4.jpg]

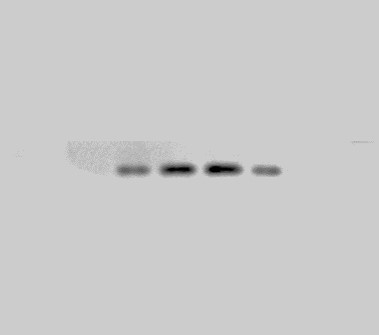

Supplement: Supplemental Information 4 [file peerj-09-11817-s004.zip › figure 3 western blot raw data/Snail.jpg]

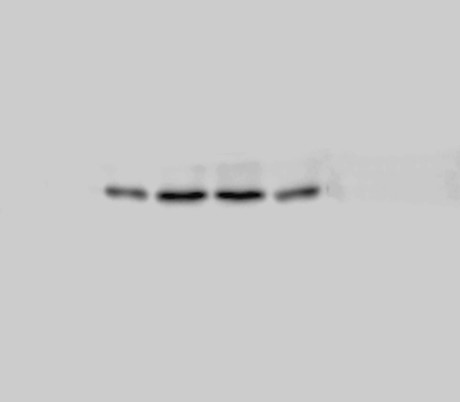

Supplement: Supplemental Information 4 [file peerj-09-11817-s004.zip › figure 3 western blot raw data/Vimentin.jpg]

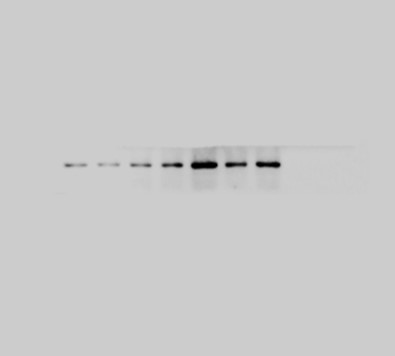

Supplement: Supplemental Information 5 [file peerj-09-11817-s005.zip › figure 5 western blot raw data/E-cadherin.jpg]

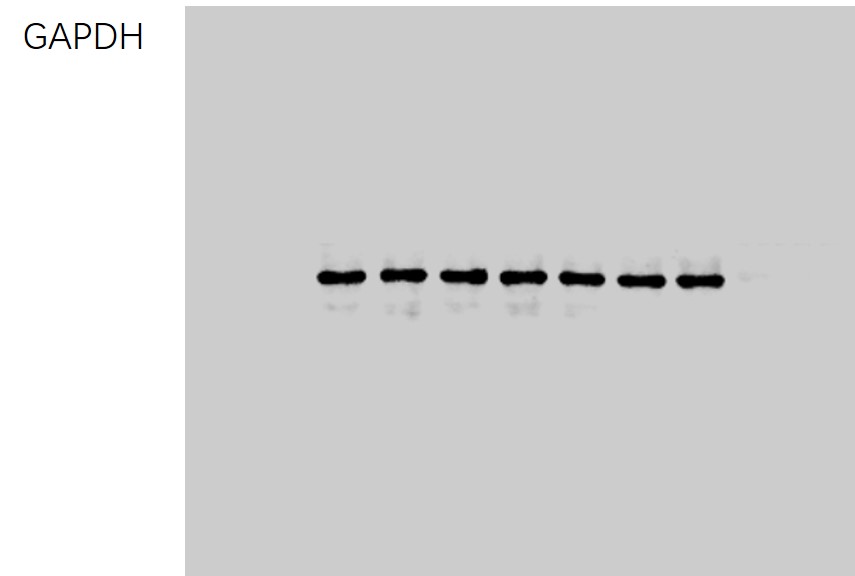

Supplement: Supplemental Information 5 [file peerj-09-11817-s005.zip › figure 5 western blot raw data/GAPDH.jpg]

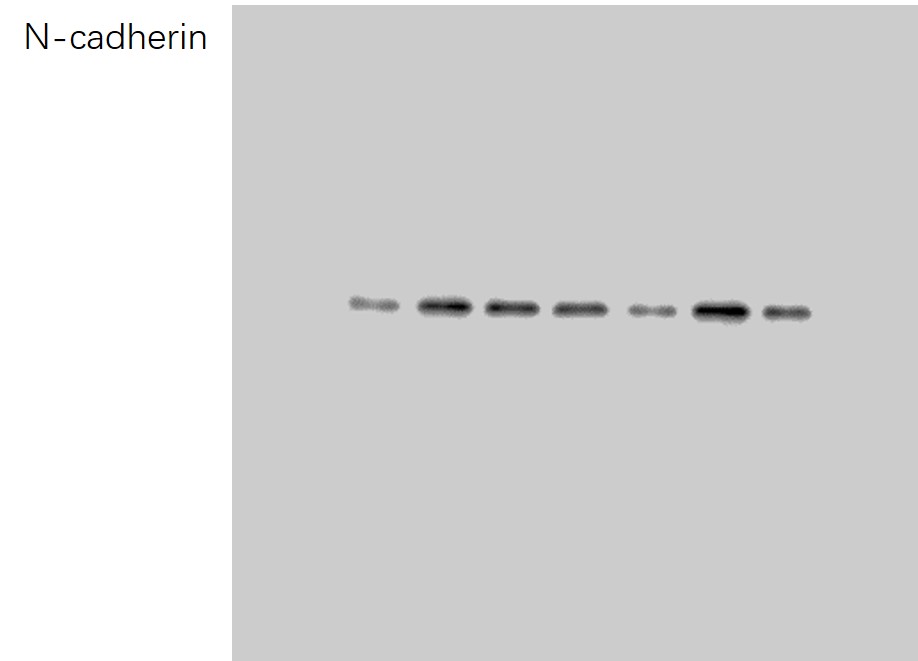

Supplement: Supplemental Information 5 [file peerj-09-11817-s005.zip › figure 5 western blot raw data/N-cadherin.jpg]

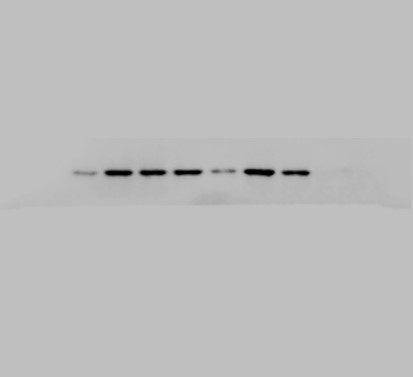

Supplement: Supplemental Information 5 [file peerj-09-11817-s005.zip › figure 5 western blot raw data/SOX4.jpg]

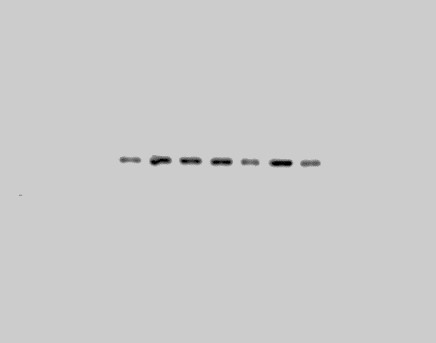

Supplement: Supplemental Information 5 [file peerj-09-11817-s005.zip › figure 5 western blot raw data/Snail.jpg]

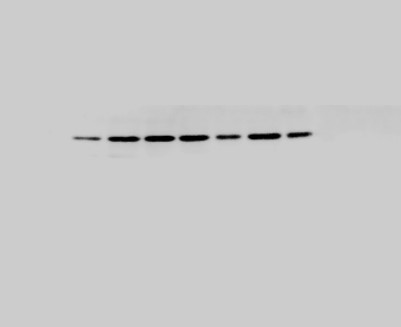

Supplement: Supplemental Information 5 [file peerj-09-11817-s005.zip › figure 5 western blot raw data/Vimentin.jpg]

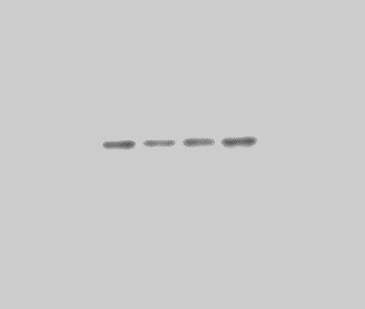

Supplement: Supplemental Information 6 [file peerj-09-11817-s006.zip › figure 6 western blot raw data/E-cadherin.jpg]

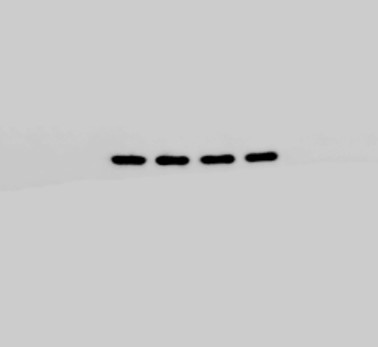

Supplement: Supplemental Information 6 [file peerj-09-11817-s006.zip › figure 6 western blot raw data/GAPDH.jpg]

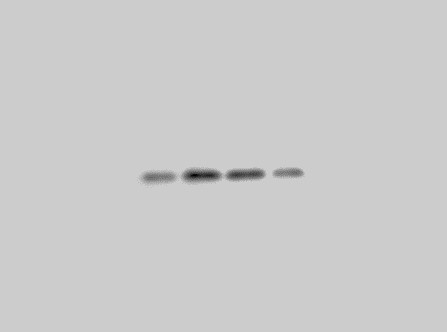

Supplement: Supplemental Information 6 [file peerj-09-11817-s006.zip › figure 6 western blot raw data/N-cadherin.jpg]

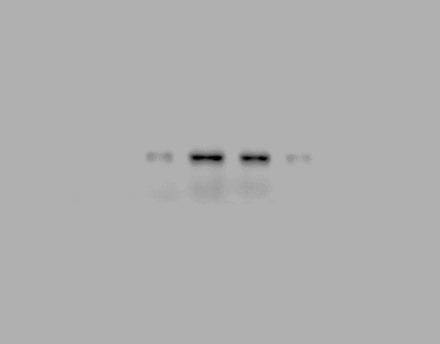

Supplement: Supplemental Information 6 [file peerj-09-11817-s006.zip › figure 6 western blot raw data/SOX4.jpg]

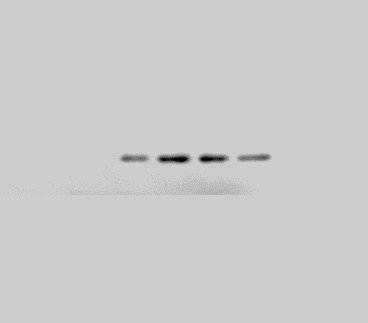

Supplement: Supplemental Information 6 [file peerj-09-11817-s006.zip › figure 6 western blot raw data/Snail.jpg]

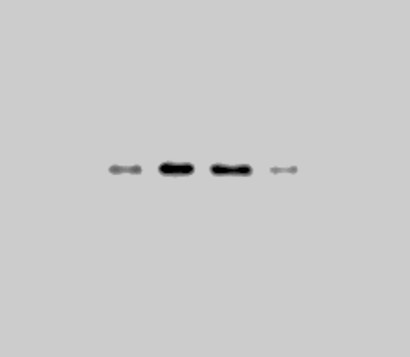

Supplement: Supplemental Information 6 [file peerj-09-11817-s006.zip › figure 6 western blot raw data/Vimentin.jpg]
